# Prognostic Significance of CRP/Albumin, D-Dimer/Albumin, D-Dimer/Fibrinogen Ratios and Triglyceride-Glucose Index in Crimean–Congo Hemorrhagic Fever: A Prospective Observational Study

**DOI:** 10.3390/tropicalmed10100287

**Published:** 2025-10-09

**Authors:** Nurten Nur Aydın, Murat Aydın

**Affiliations:** Department of Infectious Diseases and Clinical Microbiology, Erzurum Regional Training and Research Hospital, Erzurum 25080, Turkey

**Keywords:** Crimean–Congo hemorrhagic fever, D-dimer/albumin ratio, D-dimer/fibrinogen ratio, triglyceride-glucose index, mortality

## Abstract

Background: Crimean–Congo hemorrhagic fever (CCHF) is a severe zoonotic viral infection with high mortality rates. This study aimed to examine the prognostic value of new-generation inflammatory markers—CRP/albumin ratio (CAR), D-dimer/albumin ratio (DAR), D-dimer/fibrinogen ratio (DFR), and triglyceride-glucose index (TGI)—in predicting mortality among patients diagnosed with CCHF. Methods: This prospective study involved 76 patients with a positive polymerase chain reaction test for CCHF and 38 age- and sex-matched healthy controls between 15 April 2023 and 15 October 2024. Participants’ demographic, clinical, and laboratory data at presentation were recorded. Results: CAR, DAR, DFR, and TGI levels were significantly higher in the patient group compared to the control group (all *p* < 0.001). Furthermore, when mortal cases were compared with survivors, all of these markers were found to be significantly higher in the mortal group (*p* = 0.005, *p* = 0.004, *p* = 0.001, and *p* = 0.003, respectively). In Kaplan–Meier analysis, survival time was significantly shorter in patients with higher levels of these parameters (*p* < 0.001 for all). In the Receiver Operating Characteristic analysis conducted to differentiate mortal cases from survivors, DFR and TGI were identified as the markers with the highest predictive power (area under the curve: 0.938 and 0.899, respectively). Conclusions: Inflammatory markers CAR, DAR, DFR and TGI may serve as significant prognostic tools to predict mortality in CCHF.

## 1. Introduction

Crimean–Congo hemorrhagic fever (CCHF) is among the most severe vector-borne viral infections worldwide, with mortality rates of up to 30% or higher [1]. It is a zoonotic disease caused by the Crimean–Congo hemorrhagic fever virus (CCHFV), a single-stranded RNA virus belonging to the *Nairoviridae* family [2]. The disease, which exhibits seasonal increases in endemic areas, is characterized by fever, myalgia, gastrointestinal symptoms, hematological disorders, and, in severe cases, hemorrhagic manifestations [3]. The high mortality rate and rapid progression of the disease in CCHF necessitate the identification of reliable parameters that can predict prognosis early on.

Immune and inflammatory responses, such as cytokine storms, endothelial dysfunction, and coagulation disorders, play a significant role in the disease’s pathogenesis [4,5]. Therefore, in recent years, the potential roles of biomarkers reflecting systemic inflammation in infectious diseases in terms of diagnosis and prognosis have been explored [6,7,8,9]. Composite inflammatory scores (CRP/albumin ratio [CAR], D-dimer/albumin ratio [DAR], D-dimer/fibrinogen ratio [DFR], fibrinogen/albumin ratio [FAR], and triglyceride glucose index [TGI]) derived from laboratory parameters such as C-reactive protein (CRP), albumin, D-dimer, fibrinogen, triglyceride, and glucose have been reported to have prognostic value in many infectious and non-infectious clinical conditions [6,9,10,11,12]. However, the literature on the prognostic significance of inflammatory markers in patients with CCHF is limited. Specifically, there are no particular studies on DAR, DFR, or TGI. In this context, assessing the usability of new biomarkers based on readily available and routine laboratory parameters in clinical practice could greatly aid patient management and prognostication.

The aim of this study was to evaluate the prognostic significance of inflammatory markers (CAR, DAR, DFR, and TGI) in patients diagnosed with CCHF. As a secondary objective, we also aimed to evaluate survival outcomes according to marker levels.

## 2. Materials and Methods

### 2.1. Study Design and Population

This prospective, observational study was conducted at the Infectious Diseases Clinic of Erzurum Regional Training and Research Hospital, a tertiary referral center in Eastern Anatolia, Turkey, an endemic region for CCHF, from 15 April 2023 to 15 October 2024. The required sample size was determined using G*Power version 3.1.9.7 software (Heinrich-Heine-Universität Düsseldorf, Düsseldorf, Germany). For a two-sided *t*-test with a medium effect size (d = 0.65), α = 0.05, power = 0.90, and a group ratio of 2:1, a total of 114 participants (76 patients, 38 controls) were necessary. The study involved 76 patients diagnosed with Crimean–Congo hemorrhagic fever (CCHF) based on clinical and laboratory findings, along with 38 healthy volunteer controls who had similar age and gender characteristics and no known systemic disease. Patients were further classified into severe and non-severe groups based on established criteria outlined in previous studies [13,14]. Patients who met any of the following criteria within the first five days after symptom onset were considered severe: leukocyte count ≥ 10,000/mm^3^, platelet count ≤ 20,000/mm^3^, aPTT ≥ 60 s, fibrinogen ≤ 110 mg/dL, AST ≥ 200 IU/L, ALT ≥ 150 IU/L, presence of melena or hematemesis, or somnolence. Patients who did not meet these criteria were classified as non-severe. The study was carried out in line with the principles of the Declaration of Helsinki, and informed consent was obtained from all participants. Approval for the study was granted by the local ethics committee (Ethics Committee Decision No: 2022/07-68).

### 2.2. Inclusion and Exclusion Criteria

CCHF diagnosis was confirmed by real-time polymerase chain reaction (PCR) conducted on serum samples from patients at the National Reference Virology Laboratory of the Turkish Ministry of Health in cases with compatible clinical findings. Genotyping of viral strains was not carried out in this study. Individuals under the age of 18, pregnant women, those on immunosuppressive treatment, individuals with known liver or kidney failure, those with hematological diseases, and patients with another concurrent infection were excluded from the study.

### 2.3. Data Collection

Demographic and epidemiological data, including age, gender, comorbidities, occupational exposure (such as animal husbandry/farming), tick contact, and clinical symptoms, were recorded for all participants using a standardized form. Patients’ complete blood count, biochemistry, and coagulation tests performed at presentation were evaluated. Additionally, data on length of hospitalization, bleeding, and mortality were recorded. In the study, inflammatory markers CAR (CRP/albumin), DAR (D-dimer/albumin), and DFR (D-dimer/fibrinogen) values were computed. The triglyceride–glucose index (TGI) was calculated as log_10_[(triglyceride (mg/dL) × glucose (mg/dL))/2].

### 2.4. Statistical Analysis

Data were analyzed using IBM SPSS 23.0 (IBM Corp., Armonk, NY, USA) statistical package program. In the descriptive statistics of the results, the number (n) and percentage (%) were provided for categorical variables; mean and standard deviation (SD) were given for numerical variables. Two separate sets of analyses were conducted. Firstly, patients with CCHF were compared with healthy controls regarding demographic, clinical, and laboratory parameters. Secondly, within the patient group, survivors and non-survivors were compared to identify prognostic factors linked to mortality. Thirdly, patients were stratified into severe and non-severe groups based on established criteria, and these groups were compared in terms of inflammatory indices. Appropriate parametric or non-parametric tests were employed based on the distribution of the variables in each analysis. The chi-square test was employed to compare gender distribution between the patient and control groups. The normality of numerical variables was assessed with the Shapiro–Wilk test. Fibrinogen was found to follow a normal distribution and was compared using Student’s *t*-test. All other numerical variables did not follow a normal distribution and were analyzed using the Mann–Whitney U test. Bonferroni correction was used for multiple testing. For Table 1, which included six parameters, the adjusted significance level was *p* < 0.0083. For Table 2, with four parameters, the adjusted threshold was *p* < 0.0125. Receiver operating characteristic (ROC) curves were created to evaluate the discriminatory power of inflammatory markers in forecasting mortality. The area under the curve (AUC) with 95% confidence intervals was determined. The optimal threshold values were determined using Youden’s index, which identifies the cut-off point providing the best balance between sensitivity and specificity. Detailed calculation formulas and the corresponding cutt-off values for the composite indices are provided in the Supplementary Table S1. Survival times were analyzed using the Kaplan–Meier method, and groups were compared with the log-rank test. Statistical significance was set at *p* < 0.05.

## 3. Results

A total of 114 individuals participated in the study; 76 were patients with CCHF, and 38 were healthy controls. The mean age of the patients was 52.5 ± 15.2 years, similar to that of the control group at 51.1 ± 14.9 years; *p* = 0.603. No significant difference was observed between the groups regarding gender distribution (*p* = 1.000). The most common comorbidities in CCHF patients were hypertension (18.4%) and diabetes mellitus (10.5%). Tick bites were reported in 57.9% (*n* = 44) of the patients, while 59.2% (*n* = 45) were engaged in animal husbandry or farming. The most common presenting symptoms were fatigue (93.4%), fever (86.8%), myalgia/arthralgia (75.0%), headache (72.4%), nausea/vomiting (65.8%), abdominal pain (21.1%), and diarrhea (17.1%). Bleeding findings were observed in 25.0% of the patients, with the most common being epistaxis (*n* = 9; 11.8%), followed by gingival bleeding (*n* = 5; 6.6%) and gastrointestinal bleeding (*n* = 4; 5.3%). The mean length of hospital stay was 8.1 ± 3.6 days.

In the analysis of inflammatory markers, CAR, DAR, DFR, and TGI levels were found to be significantly higher in the patient group compared to the control group (all *p* < 0.001) (Table 1).

When survivors and non-survivors were compared, CAR (*p* = 0.005), DAR (*p* = 0.004), DFR (*p* = 0.001), and TGI (*p* = 0.003) values were markedly higher in the mortality group (Table 2).

When patients were stratified into severe (*n* = 32) and non-severe (*n* = 44) groups, significant differences were observed in DAR, DFR, and TGI values, all of which were markedly higher in severe cases (*p* < 0.001 for DAR and DFR, *p* = 0.001 for TGI) (Table 3).

**Figure 1:**
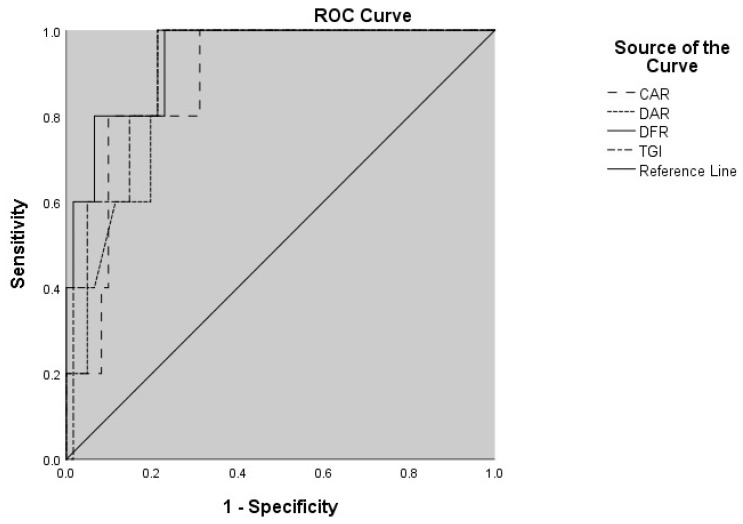
ROC curves of CAR, DAR, DFR, and TGI for predicting mortality in patients with CCHF. Abbreviations: CAR, C-reactive protein-to-albumin ratio; DAR, D-dimer-to-albumin ratio; DFR, D-dimer-to-fibrinogen ratio; TGI, triglyceride–glucose index; ROC, receiver operating characteristic; CCHF, Crimean–Congo hemorrhagic fever.

In the ROC analysis for mortality prediction, the inflammatory markers with the highest discriminatory power were DFR (AUC: 0.938; 95% CI: 0.852–1.000) and TGI (AUC: 0.899; 95% CI: 0.809–0.988), respectively (Table 4). The graphical representation of the ROC curves of the inflammatory markers is shown in Figure 1.

**Figure 2:**
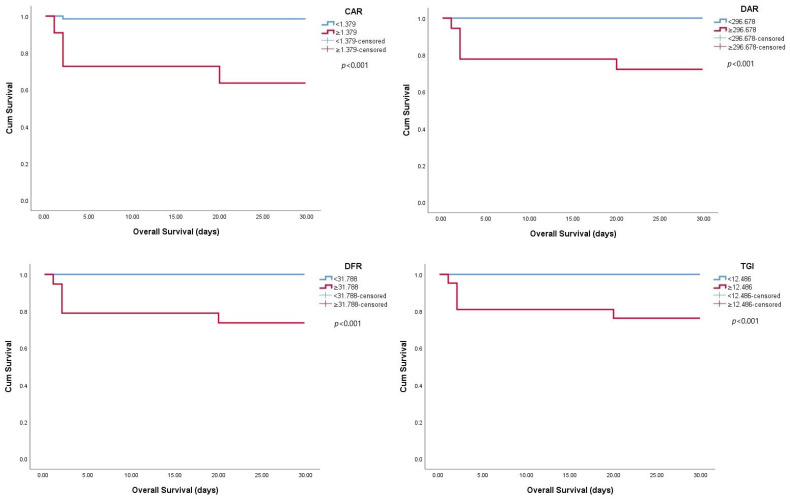
Kaplan–Meier survival curves in patients with CCHF stratified by CAR, DAR, DFR, and TGI levels. Abbreviations: CAR, C-reactive protein-to-albumin ratio; DAR, D-dimer-to-albumin ratio; DFR, D-dimer-to-fibrinogen ratio; TGI, triglyceride–glucose index; CCHF, Crimean–Congo hemorrhagic fever.

Kaplan–Meier survival analyses were conducted separately for the four markers identified as highly prognostic in ROC analysis (CAR, DAR, DFR, and TGI). These analyses showed significantly shorter survival times in patients with values exceeding the threshold values (Figure 2).

## 4. Discussion

This prospective study assessed the effect of various inflammatory markers on disease prognosis in patients diagnosed with CCHF. We first observed that CAR, DAR, DFR, and TGI levels were significantly higher in patients compared with healthy controls. Moreover, these four indices were also significantly elevated in patients who died. ROC analysis demonstrated that these four parameters possessed high discriminatory ability in predicting mortality, and survival analyses indicated that patients exceeding the threshold values had significantly shorter survival times. These results indicate that these markers could serve as potential prognostic tools for early risk stratification in CCHF patients within clinical practice.

CRP and albumin are two key biomarkers of the acute phase response, and CAR has been linked to mortality in various infections [6,15]. In our study, the CAR level was found to be significantly higher in mortal CCHF patients, and the AUC was 0.876 in ROC analysis. This finding supports earlier studies emphasizing the prognostic significance of CAR in infections such as CCHF and COVID-19 [6,16].

D-dimer is a significant biomarker indicating both coagulopathy and systemic inflammation [17,18]. Increased D-dimer levels in critically ill patients have been reported to be associated with rises in inflammatory markers such as procalcitonin and interleukin-6, supporting the interaction between inflammation and coagulation dysfunction [17]. It has been reported that derived ratios such as DAR and DFR are more effective determinants for predicting the prognosis of infections than traditional parameters, particularly in studies involving COVID-19 patients [19,20]. While D-dimer is regarded as an indicator of intravascular coagulation and fibrinolytic activity by reflecting fibrin degradation products, fibrinogen is an acute phase reactant that rises during inflammation and can be consumed and decreased in severe cases developing disseminated intravascular coagulation [21,22]. Therefore, DFR can be regarded as a composite indicator of mechanisms such as endothelial damage, systemic inflammation, coagulopathy, and consumption coagulopathy that may be observed in CCHF. Indeed, in our study, both DAR and DFR levels were found to be significantly higher in the mortal group; in the ROC analysis, these two parameters stood out with high AUC values of 0.892 and 0.938, respectively.

TGI has been defined as a metabolic marker reflecting insulin resistance and has been investigated in infectious diseases in recent years due to its association with systemic inflammation, endothelial dysfunction, and metabolic stress [23,24,25,26]. In the pathogenesis of viral infections, metabolic responses such as decreased insulin sensitivity, increased hepatic gluconeogenesis, and accelerated lipolysis are involved under the influence of proinflammatory cytokines (e.g., TNF-α, IL-6) [27,28,29,30]. TGI, as a combined indicator of glucose and triglyceride levels, reflects both increased glucose caused by proinflammatory cytokines and hypertriglyceridemia resulting from the activation of lipolysis. Therefore, it can be argued that the TGI level reflects not only systemic inflammation but also the metabolic response associated with the severity of the disease. In our study, the TGI level was found to be significantly higher in mortal cases and emerged as a strong prognostic indicator for predicting mortality, with an AUC value of 0.899 in ROC analysis.

The prognostic significance of these indices may be due to their reflection of underlying metabolic and coagulation disturbances in CCHF. Elevated glucose and triglyceride levels indicate stress hyperglycemia and altered lipid metabolism, both of which have been linked to systemic inflammation, endothelial dysfunction, and adverse outcomes in viral infections [24,26]. Albumin, as a negative acute-phase reactant, decreases in severe disease, and its inclusion in CAR and DAR further enhances their prognostic value [6,16,19]. Conversely, increased D-dimer levels alongside decreased fibrinogen levels indicate activation of coagulation and secondary fibrinolysis, aligning with the hemorrhagic tendency of CCHF [31,32]. Thus, indices such as DAR and DFR measure the extent of coagulation disturbances, while TGI combines metabolic dysregulation, offering a wider view of the host response to infection. These mechanisms might explain the strong link between these indices and mortality seen in our cohort. For potential clinical use, the optimal cut-off values determined by ROC analysis were 1.379 for CAR (sensitivity 80.0%, specificity 90.1%), 296.678 for DAR (sensitivity 100.0%, specificity 79.0%), 31.788 for DFR (sensitivity 100.0%, specificity 77.0%), and 12.486 for TGI (sensitivity 100.0%, specificity 77.5%). These thresholds may help guide early risk stratification in CCHF patients.

This study has several limitations. First, because it was conducted at a single center with a small number of participants, the generalizability of the findings is limited. Furthermore, since the study was conducted in Eastern Anatolia, Turkey, an endemic region for CCHF, the findings may be affected by regional epidemiological features, such as viral strain differences and population-specific factors, and may not be fully applicable to other geographical areas. Particularly, including only five patients in the mortality subgroup raises the risk of bias in statistical analyses and restricts the assessment of prognostic biomarkers’ performance. Although the inflammatory indices used in the study are parameters that reflect dynamic processes, they were calculated using data obtained only from a single time point at admission; this prevents the assessment of changes in biomarkers over time and their relationship with the clinical course. Another limitation is that, although none of the healthy controls had comorbidities, some patients did (e.g., hypertension, diabetes mellitus). Therefore, we cannot completely rule out the possibility that some of the differences observed in laboratory parameters, especially metabolic markers like glucose and triglycerides, may have been affected by these conditions rather than by CCHF alone. Furthermore, body mass index (BMI) data were not gathered, which further hampers our capacity to evaluate potential confounding by metabolic status. Although we analyzed each parameter independently, advanced multivariate or machine learning approaches might further clarify the interrelationships between biomarkers in CCHF. Future studies with larger datasets could incorporate such methods to offer additional insights.

In conclusion, this study showed that composite inflammatory markers such as CAR, DAR, DFR, and TGI may possess significant prognostic value in predicting mortality in CCHF patients. These readily available biomarkers from standard laboratory tests may assist in the early detection of high-risk patients when incorporated into clinical practice.

## Figures and Tables

**Table 1:** Comparison of demographic, clinical and laboratory findings of CCHF patients and healthy individuals.

Variables	Patients (*n* = 76)	Controls (*n* = 38)	*p*-Value
Age (Years) ± SD	52.5 ± 15.2	51.1 ± 14.9	0.603
Gender Male, n (%) Female, n (%)	33 (43.4%) 43 (56.6%)	16 (42.1%) 22 (57.9%)	1.000
Comorbidities			
Hypertension	14 (18.4%)	0 (0%)	
Diabetes mellitus	8 (10.5%)	0 (0%)	
Cardiovascular disease	6 (7.9%)	0 (0%)	
Chronic obstructive lung disease	1 (1.3%)	0 (0%)	
Laboratory findings			
CAR	0.68 ± 0.80	0.06 ± 0.02	<0.001
DAR	299.76 ± 390.92	5.14 ± 1.17	<0.001
DFR	48.38 ± 71.49	0.81 ± 0.23	<0.001
TGI	11.44 ± 15.28	3.01 ± 0.97	<0.001

Bonferroni-adjusted significance threshold for Table 1: *p* < 0.0083. *p*-values were calculated using the Mann–Whitney U test. Abbreviations: CAR, CRP-to-albumin ratio; DAR, D-dimer-to-albumin ratio; DFR, D-dimer-to-fibrinogen ratio; TGI, triglyceride-glucose index.

**Table 2:** Comparison of laboratory parameters between survivors and non-survivors among CCHF patients.

Laboratory Findings	Survivor (*n* = 71)	Non-Survivor (*n* = 5)	*p*-Value
CAR	0.59 ± 0.66	1.95 ± 1.52	0.005
DAR	235.59 ± 358.16	872.25 ± 350.07	0.004
DFR	37.13 ± 55.36	185.64 ± 107.65	0.001
TGI	10.61 ± 15.23	23.22 ± 11.50	0.003

Bonferroni-adjusted significance threshold for Table 2: *p* < 0.0125. *p*-values were calculated using the Mann–Whitney U test. Abbreviations: CAR: CRP-to-albumin ratio; DAR: D-dimer-to-albumin ratio; DFR: D-dimer-to-fibrinogen ratio; TGI: Triglyceride-glucose index.

**Table 3:** Comparison of laboratory parameters between non-severe and severe CCHF patients.

Laboratory Findings	Non-Severe (*n* = 44)	Severe (*n* = 32)	*p*-Value
CAR	0.47 ± 0.48	0.97 ± 1.05	0.052
DAR	92.27 ± 143.49	571.63 ± 444.99	<0.001
DFR	11.30 ± 16.02	98.71 ± 85.96	<0.001
TGI	7.31 ± 4.88	17.11 ± 21.78	0.001

Bonferroni-adjusted significance threshold for Table 3: *p* < 0.0125. *p*-values were calculated using the Mann–Whitney U test. Abbreviations: CAR: CRP-to-albumin ratio; DAR: D-dimer-to-albumin ratio; DFR: D-dimer-to-fibrinogen ratio; TGI: Triglyceride-glucose index.

**Table 4:** ROC analysis of inflammatory markers for predicting mortality in CCHF patients.

Variables	AUC (95% CI)	Cut-Off Value	*p*-Value	Sensitivity (%)	Specificity (%)
CAR	0.876 (0.761–0.991)	1.379	0.005	80.0	90.1
DAR	0.892 (0.798–0.986)	296.678	0.004	100.0	79.0
DFR	0.938 (0.852–1.000)	31.788	0.001	100.0	77.0
TGI	0.899 (0.809–0.988)	12.486	0.003	100.0	77.5

Abbreviations: AUC: Area under the curve; CAR: CRP-to-albumin ratio; DAR: D-dimer-to-albumin ratio; DFR: D-dimer-to-fibrinogen ratio; ROC: Receiver operating characteristic; TGI: Triglyceride-glucose index.

## Data Availability

The data presented in this study are available on request from the corresponding author. (please specify the reason for restriction, e.g., the data are not publicly available due to privacy or ethical restrictions.)

## Supplementary Materials

The following supporting information can be downloaded at: https://www.mdpi.com/article/10.3390/tropicalmed10100287/s1, Table S1: Threshold values and calculation formulas of composite indices.
