# Supplementary material for: Prognostic Significance of CRP/Albumin, D-Dimer/Albumin, D-Dimer/Fibrinogen Ratios and Triglyceride-Glucose Index in Crimean–Congo Hemorrhagic Fever: A Prospective Observational Study
Source: Trop Med Infect Dis. 2025 Oct 9;10(10):287. doi: 10.3390/tropicalmed10100287 (PMC12568074; doi:10.3390/tropicalmed10100287)
Supplement: Supplementary file 1 [file tropicalmed-10-00287-s001.zip › tropicalmed-3817671-supplementary.pdf]

Supplementary

Table S1. Threshold values and calculation formulas of composite indices

Index	Formula	Cut-off value (mortality prediction)	Sensitivity (%)	Specificity (%)	Interpretation
CAR	CRP (mg/L) / Albumin (g/L)	1.379	80.0	90.1	Higher CAR indicates increased mortality risk
DAR	D-dimer (ng/mL) / Albumin (g/L)	296.678	100.0	79.0	Higher DAR indicates increased mortality risk
DFR	D-dimer (ng/mL) / Fibrinogen (mg/dL)	31.788	100.0	77.0	Higher DFR indicates increased mortality risk
TGI	Log_{10} [Triglyceride (mg/dL) × Glucose (mg/dL) / 2]	12.486	100.0	77.5	Higher TGI indicates increased mortality risk

Abbreviations: AUC: Area under the curve; CAR: CRP-to-albumin ratio; DAR: D-dimer-to-albumin ratio; DFR: D-dimer-to-fibrinogen ratio; ROC: Receiver operating characteristic; TGI: Triglyceride-glucose index.
